# Controlling Chloride Crossover in Bipolar Membrane Water Electrolysis

**DOI:** 10.1021/acselectrochem.5c00175

**Published:** 2025-07-16

**Authors:** Maria F. Rochow, Daniela H. Marin, Harrison J. Cassady, Ryan T. Hannagan, Katherine Yan, Joseph T. Perryman, Adam C. Nielander, Thomas F. Jaramillo, Michael A. Hickner

**Affiliations:** † Department of Material Science and Engineering, The Pennsylvania State University, University Park, Pennsylvania 16802, United States; ‡ Department of Chemical Engineering, 6429 Stanford University, Stanford, California 94305, United States; § SUNCAT Center for Interface Science and Catalysis, SLAC National Accelerator Laboratory, Menlo Park, California 94025, United States; ∥ Department of Chemical Engineering, The Pennsylvania State University, University Park, Pennsylvania 16802, United States

**Keywords:** Bipolar membranes, Ion transport, Ion crossover, Water electrolysis, Seawater

## Abstract

Bipolar membranes (BPMs) are increasingly recognized as a promising electrolyte option for water electrolysis, attributable to their distinctive properties derived from the membrane’s layered structure, which consists of an anion exchange (AEL) and a cation exchange layer (CEL). This study investigates four different BPMs and the influence they have on the performance of a water electrolysis cell under two different feed configurations: (1) a symmetric deionized water feed to both anode and cathode compartments and (2) an asymmetric feed with a 0.5 mol/L NaCl catholyte feed and a deionized water anolyte feed. The BPMs were also investigated for total chlorine (Cl) species (e.g., Cl^–^, Cl_2_, HOCl, and ClO^–^) in the anolyte due to Cl^–^ crossover from the catholyte during water electrolysis with the asymmetric feed, at an applied current density of 250 mA/cm^2^. The best-performing BPM with the asymmetric feed was an E98-05 (CEL)/FAS-50 (AEL) membrane with a TiO_2_ water dissociation catalyst at the BPM junction. This membrane had the lowest measured Cl species crossover and lowest cell voltage at a given current density under asymmetric conditions compared to the other BPMs studied. It was also found that under asymmetric conditions the CEL facing the catholyte feed determined the amount of total Cl species crossover due to anion exclusion (Donnan exclusion) of the CEL, reducing the amount of Cl^–^ in the CEL where it crossed over to the AEL and the anolyte compartment.

## Graphical abstract

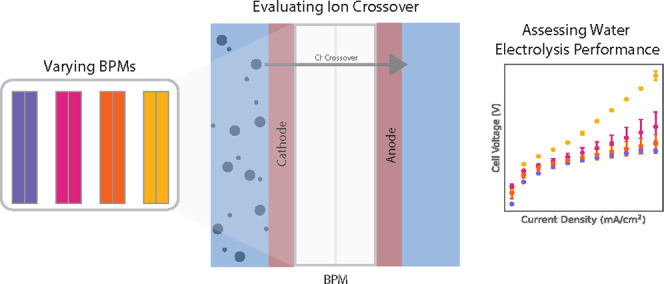

## Introduction

Hydrogen (H_2_) is increasingly becoming a cornerstone of the global energy landscape, particularly in the swift transition towards renewable energy sources, low-carbon fuels, and the technologies that support them. As of 2021, most H_2_ is derived from fossil fuels, with around 50 % of global production originating from natural gas.^1,2^ One prevalent method for H_2_ production, steam methane reforming, results in the emission of 7.5 to 12 tons of CO_2_ for every ton of H_2_ produced.^3^ This large environmental impact underscores the urgent need to explore alternative H_2_ production methods that minimize their reliance on fossil fuels.

Water electrolysis presents a promising pathway for sustainable hydrogen (H_2_) production, utilizing electrical energy to split water molecules into H_2_ and O_2_:^4^

1 $$2H_{2}O_{\left(l\right)}\to 2{H_{2}}_{\left(g\right)}+{O_{2}}_{\left(g\right)}$$

When powered by renewable electricity, this process enables an environmentally friendly method of generating hydrogen fuel with minimal carbon emissions.^5^

The operation of commercial membrane electrolyzers requires high-purity water with a resistivity greater than 1 MΩ cm and a Na^+^ and Cl^–^ content below 5 μg/L.^6^ Although producing large volumes of such high-purity water increases both capital and operational expenditures, electricity remains the dominant contributor to the levelized cost of hydrogen production in water electrolysis systems.^7,8^

Recent efforts have aimed to reduce the barriers to H_2_ production using electrolysis, such as alternative feed sources and novel electrolyzer assemblies.^9,10^ Concerning alternative feed sources, the widespread availability of seawater, making up 96.5 % of Earth’s water, together with its suitability for direct electrolysis presents a strong case for its potential adoption.^11,12^

Yet, there are challenges with direct seawater electrolysis, including the high concentrations and wide variety of ions present in seawater.^13^ For example, the Mg^2+^ ion can block active sites on the catalyst surfaces at the cathode electrode.^14^ The Cl^–^ ions can oxidize at the anode, giving undesirable side products:^14,15^

2 $$2Cl\to {Cl}_{2}+2e^{-}$$

3 $${Cl}^{-}+2OH\to {ClO}^{-}+H_{2}O+2e^{-}$$

Cl_2_ evolution (eq 2, at low pH) and hypochlorite formation (eq 3, at high pH) compete with the oxygen evolution reaction (OER) at the anode, decreasing both the electrochemical performance and durability of the electrolyzer.^16,17^

To minimize the detrimental effects of the Cl^–^ oxidation reactions, research efforts have focused on synthesizing selective and durable catalysts for the OER, as well as investigating different water electrolysis cell and feed configurations (i.e. symmetric versus asymmetric).^18,19^ These configurations include operation in a liquid alkaline electrolyte (alkaline water electrolysis), systems utilizing either a proton (PEM) or anion exchange membrane (AEM), or high-temperature cells with a proton-conducting ceramic separator.^14,18,20–22^ Marin et al.^20^ demonstrated that an asymmetric feed (0.5 mol/L NaCl as the catholyte feed and deionized water as the anolyte feed) in conjunction with a bipolar membrane (BPM) greatly suppresses the Cl^–^ oxidation reaction, through reduced Cl^–^ concentrations and more basic local microenvironment at the anode.

A BPM is a layered structure comprised of a cation exchange layer (CEL) and an anion exchange layer (AEL).^23^ In BPM water electrolysis, a voltage is applied across a BPM, and electro-dissociation of water occurs at the junction between the CEL and AEL, typically aided by a water-dissociation catalyst, producing H^+^ and OH^–^ ions.^24,25^ From the junction, H^+^ ions move through the CEL, while OH^–^ ions move through the AEL. H_2_ gas is produced at the cathode and O_2_ gas is produced at the anode.^26,27^

Since Cl^–^ crossover negatively impacts the performance of direct seawater electrolysis, optimizing the design of BPMs to minimize this effect is crucial. This study evaluates the performance of various ion exchange layers, specifically focusing on reducing Cl^–^ crossover in an operating electrolyzer. The report details the performance of four BPMs during water electrolysis under two different feed conditions: 1) a symmetric deionized water feed for both electrolytes, and 2) an asymmetric electrolyte feed with 0.5 mol/L NaCl as the catholyte, and deionized water as the anolyte (Figure 1). The electrochemical performance was assessed by examining polarization curves from electrolyzer experiments, and the influence of membrane properties on the electrochemical performance will be discussed. Furthermore, ion crossover in these systems is analyzed using ICP-MS and pH measurements. Finally, the study investigated which layer, either the CEL or AEL, has a greater influence on Cl^–^ crossover. This study offers an important contribution to the literature by systematically evaluating four distinct BPM combinations in an important electrolyzer configuration. It directly investigates the extent to which each BPM configuration influences Cl^–^ crossover and overall electrolyzer performance, an area not thoroughly addressed in prior work. The findings provide new insights into BPM design and ion transport dynamics and significantly advance the understanding of BPM behavior in water electrolyzer systems.

**Figure 1:**
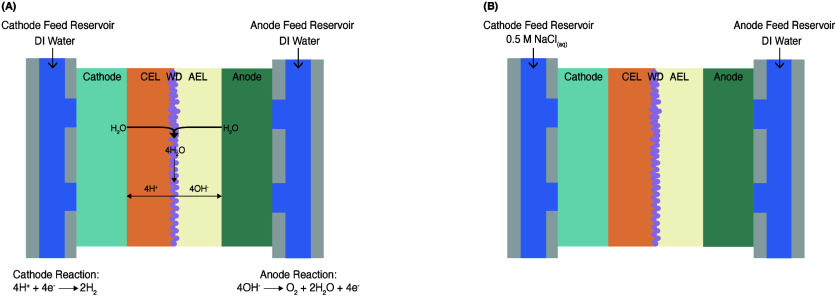
Feed conditions during BPM water electrolysis: (A) symmetric feed with deionized water for both electrolytes and (B) asymmetric feed with DI water at the anolyte and 0.5 mol/L NaCl as the catholyte feed.

## Materials and methods

For a detailed explanation of the methodologies and procedures used in this study, readers are encouraged to consult the works of Rios Amador et al.^28^ and Marin et al.^20^

### Anode Fabrication

Anodes used for the BPM water electrolysis testing were fabricated by spraying IrO_*x*_ catalyst ink onto a platinized Ti porous transport layer substrate. The catalyst ink was produced by combining 0.1 g of IrO_*x*_ (The Fuel Cell Store, Product Code: 11080001), which had a high BET reported surface area of 110 m^2^/g to 130 m^2^/g and average crystallite size of 3 nm to 4 nm, 0.47 g of ultrapure deionized water (Millipore Sigma, Milli-Q IQ 7000 Ultrapure Water Purification System), 1.7 g of isopropyl alcohol, and 0.1 g of 5 wt % of PiperION ionomer solution (The Fuel Cell Store, Product Code: 11080001).^29^ This mixture was then sonicated in a bath sonicator (Branson) for 20 min until minimal catalyst agglomerates were visible.

A 5 cm × 5 cm platinized Ti fiber felt with a thickness of 0.01 in (The Fuel Cell Store, Product Code: 40050006) was used as the porous transport layer. The catalyst ink was sprayed onto the porous transport layer with a hand-held airbrush (Testors) until the catalyst reached the desired mass loading of 2.9 mg/cm^2^. Loading was determined by intermittent weighing of the porous transport layer during catalyst ink spraying.

An ionomer overlayer was sprayed on top of the catalyst layer using a 2.5 wt % PiperION ionomer (A40) (Fuel Cell Store, Product Code: 11080001) diluted with ethanol. This PiperION solution was sprayed in a serpentine pattern with the airbrush over the catalyst layer until the ionomer solution comprised between 10 % to 20 % of the catalyst weight. The anodes were placed and stored in Petri dishes at room temperature until use.

### Cathode Fabrication

Cathodes used for BPM water electrolysis testing were fabricated by spraying 5 % platinum on carbon catalyst ink onto a carbon paper porous transport layer substrate. The catalyst ink was produced by combining 0.1 g of 5 % platinum on Carbon Vulcan XC-72 catalyst (The Fuel Cell Store, Product code: 57080184), 1.5 g of ultrapure deionized water, 1.7 g of isopropyl alcohol, and 0.1 g of 5 wt % of Nafion ionomer (Ion Power).^30^ This mixture was then bath sonicated for 30 min until minimal catalyst agglomerates were visible.

A 5 cm × 5 cm Toray carbon paper, wet-proofed with a thickness of 0.37 mm was used as the porous transport layer (The Fuel Cell Store, Product Code: 71070043). The catalyst ink was sprayed onto the porous transport layer with a hand-held airbrush until the catalyst reached the desired loading of 2.0 mg/cm^2^.

An ionomer overlayer was sprayed on top of the catalyst layer using a 5 wt % Nafion D520 ionomer solution dispersed in water and isopropanol (Ion Power). This solution was sprayed in a serpentine pattern over the catalyst layer until the ionomer solution comprised between 10 % to 20 % of the catalyst layer weight. When dry, the cathodes were placed and stored in Petri dishes at room temperature until use.

### Bipolar Membrane Fabrication

#### Membranes

Building on previous work that evaluated component membranes based on NaCl flux and area resistance, four BPMs (CEL/AEL) were fabricated for this study.^31^ A selection approach was developed with a focus on transport properties as the primary criterion for choosing suitable component membranes (CEL and AEL). The four BPMs were: Aquivion E98-05/Fumasep FAS-50 (E98-05/FAS-50), Fumasep FS-720/PiperION (FS-720/A40), Nafion 212/Fumasep FAA-3-50 (N212/FAA-3-50), N212/PiperION (N212/A40). All membranes were purchased from The Fuel Cell Store, except for N212 which was purchased from Ion Power. The thicknesses of each membrane were measured in the dry state using a digital micrometer (Mitutoyo 293-831-30, Kawasaki, Japan) (Table 1). Before fabrication, all membranes were pretreated per manufacturer recommendations. N212 was placed in boiling water for 1 h for pretreatment, which is standard practice.^32^ To ensure the AEMs were in OH^–^ form, they were placed into a 1 mol/L KOH solution for 24 h, replacing the solution three times. After pretreatment, membranes were stored in DI water until use.

**Table 1:** Information for Membranes Used in the Study

Membrane	Type	Chemical Structure^a^	Thickness^b^ (μm)	IEC^c^ (meq/g)
Fumasep FAA-3-50	AEM	HC	32	1.6–2.1
Fumasep FAS-50	AEM	HC	53	1.6–2.0
PiperION (A40)	AEM	HC	40	2.35
Aquivion E98-05	CEM	SSC-PFSA	50	>1.0
Nafion 212	CEM	LSC-PFSA	50	0.95–1.01
Fumasep FS-720	CEM	PFSA	15	1.39

a The chemical structure of the membranes is classified into several types: hydrocarbon based (HC), short-side chain perfluorosulfonic acid (SSC-PFSA), long-side chain perfluorosulfonic acid (LSC-PFSA), and perfluorosulfonic acid (PFSA) based.

b Measured as received in the dry membrane state.

c As listed on the membrane data sheet.

### Water Dissociation Catalyst Layer

The water dissociation layer (WD) is a catalyst layer at the interface of the AEL and CEL of the BPM to facilitate water splitting. The catalyst ink consisted of 0.1 g of Aeroxide TiO_2_ P25 nanoparticles with a reported specific surface area (BET) of 35 m^2^/g to 65 m^2^/g (Nippon Aerosil Co., Ltd), and 4.9 g of DI water.^33^ The ink was then placed in an ultrasonic bath until fully dispersed (as evident by the lack of visible catalyst aggregates). Then 30 mg of the concentrated stock solution was diluted in 0.47 g of water and 1.7 g of isopropyl alcohol. This newly created ink was sonicated for 5 min before spraying. The water dissociation layer was applied by placing a 1.5 cm × 1.5 cm CEM on a 90 °C heated bed and by hand-spraying an area 1.1 cm × 1.1 cm. After the application of the water dissociation catalyst layer, the CEM was stored in deionized water until BPM fabrication.

### Bipolar Membrane Fabrication

BPM fabrication occurred immediately before testing in the electrolyzer. The BPM was fabricated by placing the CEL on a glass plate with the WD layer facing up. The AEL was then rolled onto the CEL making sure that no bubbles were trapped between the layers. The BPM was then placed directly into the electrolyzer.

### Electrolyzer Assembly

The electrolyzer hardware used for all experiments was from Fuel Cell Technologies, Inc. (5 cm^2^ Cell Hardware, Product Code: 5SCH). The electrolyzer used a Ti flow field on the anode side and a graphite flow field on the cathode side. A sintered Ti separator (400 μm, Baoji Yinggao Metal Materials Inc.) was placed between the flow fields and each electrode. The membrane area was screened to 1 cm × 1 cm using a series of PET gaskets, with the gaskets having a total thickness of 0.037 in on both anode and cathode sides.

### Cell Operation Parameters

For the liquid electrolytes, two borosilicate glass reservoirs contained DI water (1 L of DI water per container) and were heated to 60 °C with constant stirring. The anolyte and catholyte were pumped through the electrolyzer at flow at a rate of 60 mL/min before being returned to the reservoir. The flow rate was verified with a graduated cylinder and stopwatch before the experiment. Before electrolyzer operation, the internal electrolyzer assembly’s temperature was monitored until the temperature reached and stabilized at 50 °C via a thermocouple mounted in the electrolyzer endplate.

Stepped chronopotentiometry measurements were performed using a Biologic potentiostat (Seyssinet-Pariset, France). The current was increased from 50 to 500 mA/cm^2^ in 50 mA/cm^2^ increments, holding for 1 min at each step. At 500 mA/cm^2^, the current was held for 10 min to ensure baseline electrolyzer performance.^20^ The current was then decreased from 500 to 50 mA/cm^2^ in 50 mA/cm^2^ increments, holding for 1 min at each step. Finally, the current was increased again from 50 to 500 mA/cm^2^ in 50 mA/cm^2^ increments, holding for 10 s at each step. Data for the DI water polarization curves were extracted from the last set of stepped chronopotentiometry measurements.

Galvanostatic electrochemical impedance spectroscopy (GEIS) measurements took place at the following specified current densities: 500, 250, 100, and 50 mA/cm^2^. The AC oscillation frequency was scanned from 600 Hz to 20 kHz. The amplitude of the frequency changed for each current density, where the 500 mA/cm2 had an amplitude of 30 mA, 250 mA/cm^2^ had an amplitude of 10 mA, 50 mA/cm^2^ had an amplitude of 3 mA, and 10 mA/cm^2^ had an amplitude of 1 mA.

Following the GEIS measurements, the current density was held at 250 mA/cm^2^ until the voltage response reached a steady state; then NaCl was added to achieve a 0.5 mol/L NaCl solution in the catholyte reservoir. A pH probe (Thermo Scientific Orion 8102BNUWP ROSS Ultra pH Electrode), which was connected to a Thermo Fisher Scientific (Waltham, MA) OrionStar A212 meter, was inserted into the anolyte reservoir during the asymmetric feed experiments to measure the change and pH as a function of time. To understand ion concentrations and ion transport within this electrolyzer system, pH was converted into H^+^ concentration. The H^+^ accumulation rate was then calculated from the slope of the H^+^ concentration versus time. A DC current of 250 mA/cm^2^ was then held for 2 h, during which 8 mL aliquots were taken from the anolyte reservoir every 30 min. The aliquots were acidified with 0.5 mL (per aliquot) of 70 % nitric acid (Fisher Chemical, TraceMetal Grade) for ICP-MS analysis.

Finally, stepped chronopotentiometry measurements were performed again with the same parameters as the initial stepped chronopotentiometry sequence, however this time with the catholyte containing the 0.5 mol/L NaCl solution. Data for the 0.5 mol/L NaCl polarization curves were extracted from the last chronopotentiometry measurements. GEIS was then performed using the same parameters as the initial GEIS sequence. The HFR measurements were taken immediately after the polarization curve to account for any changes with the membrane that may have occurred during the 195 min current hold and to ensure steady-state transport. To determine the BPM resistance from the GEIS data via high-frequency resistance (HFR) measurements, a semicircular fit was applied to the high-frequency region of the Nyquist plot and extrapolated to its intersection with the real axis. HFR values and BPM resistance calculated at a constant current density of 250 mA/cm^2^, during both feed conditions (symmetric and asymmetric).

Each electrolyzer experiment was conducted a minimum of three times to obtain an estimate of the uncertainty of the measurement. The error bars represent one standard deviation from the mean.

### ICP-MS Measurements

Autosampler measurements for trace-elemental analysis were conducted using a Thermo Scientific iCAP RQ ICP-MS with a parallel flow nebulizer (Burgener PEEK Mira Mist). The nebulizer flow rate was 1.15 L/min. The Peltier-cooled quartz cyclonic spray chamber was set to 2.7 °C. Platinum sample and skimmer cones were used (ThermoFisher Scientific), along with a 3.5 mm high matrix insert (Thermo Fisher Scientific). The ICP-MS operated in standard mode to detect ^35^Cl and ^37^Cl. The plasma power was set to 1550 W. The aliquots were each sampled three times using a dwell time of 0.1 s, with each repetition consisting of 20 sweeps. Cl^–^ standard solution was prepared for a concentration range from 0.1 ppm to 5 ppm using serial dilution of a NaCl standard using 2 % nitric acid (Sigma-Aldrich, TraceCERT, 1 g/L Na in nitric acid).

## Results and Discussion

To elucidate the performance metrics of the BPM water electrolyzer, the cell voltage was measured as a function of increasing current density. Two different feed conditions were considered to determine the effects of impure electrolyte feeds with different BPMs: (1) a *symmetric* feed, where both the catholyte and anolyte feeds were DI water, and (2) an *asymmetric* feed, where the catholyte feed was 0.5 mol/L NaCl solution and the anolyte feed was DI water. The asymmetric configuration was designed to mitigate Cl^–^ oxidation at the anode by using the BPM’s selective ion transport properties: the CEL limits Cl^–^ crossover, while the AEL creates a locally alkaline environment that suppresses chloride oxidation in favor of oxygen evolution.

The polarization curves of the four BPMs under both symmetric and asymmetric feed conditions are shown in Figure 2. Figure 2 shows that the asymmetric feed resulted in a higher cell voltage compared to the symmetric DI water feed for all membranes tested.

**Figure 2:**
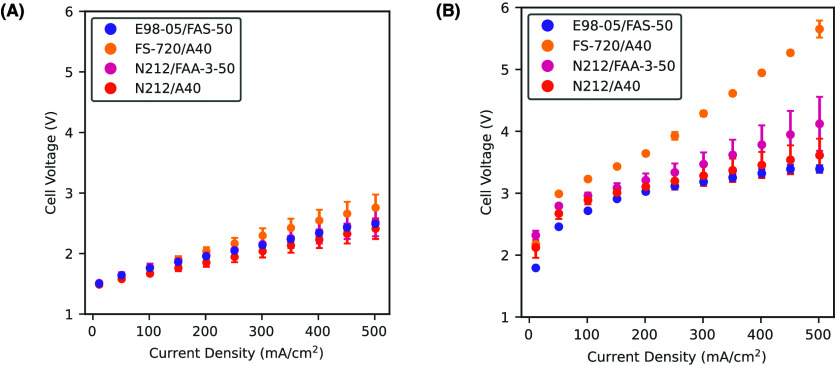
Polarization curves produced during BPM water electrolysis for characterization of device performance. Curves were produced at two different catholyte feed conditions: (A) DI water (symmetric feed) and (B) 0.5 mol/L NaCl solution (asymmetric feed), both anolyte feeds were DI water.

For the symmetric feed, the BPMs from lowest to highest voltage are N212/A40, E98-05/FAS-50, N212/FAA-3-50, and FS-720/A40, at current densities greater than 150 mA/cm^2^. With the asymmetric feed, this order changed to E98-05/FAS-50, N212/A40, N212/FAA-3-50, and FS-720/A40 (at current densities greater than 10 mA/cm^2^).

Ions in seawater, such as Na^+^ and Cl^–^ ions, adversely affect electrolyzer performance by contributing to resistance and activation overpotentials resulting in elevated cell voltages.^34,35^ This contaminant effect is observed in the elevated cell voltages that occur with the asymmetric feed versus the symmetric feed. This analysis will primarily concentrate on the overpotentials associated with the BPM, specifically the ohmic losses. However, the observed shift in the slope of the FS-720/A40 polarization curve in Figure 2(B), beginning at approximately 250 mA/cm^2^, may suggest the onset of mass transport losses contributing to increased overpotentials, relative to the other three BPM polarization curves.

The HFR of the bipolar membranes (at a fixed current density of 250 mA/cm^2^) in the electrolysis device for (DI water) symmetric and (NaCl) asymmetric feeds is shown in Figure 3. The HFR provides the ohmic resistance of the system, of which the membrane resistance is a major component. Since the electrolyzer setup is the same, it will be assumed that changes in the ohmic resistance are due to differences with the BPM layers.

**Figure 3:**
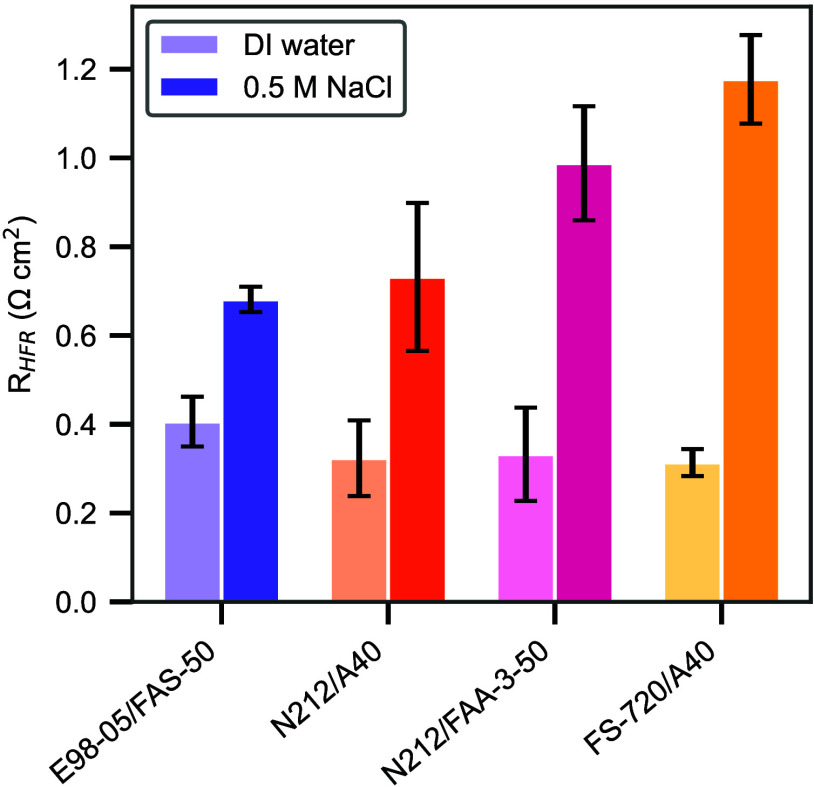
High-frequency resistance values, from GEIS measurements, for each of the four different BPM tested, for the two different catholyte feed conditions: Symmetric (left bar) and asymmetric (right bar). The resistance values were measured at a fixed current density of 250 mA/cm^2^.

At 250 mA/cm^2^, we observe a 0.5 Ω cm^2^ difference in resistance between the BPM with the lowest HFR, E98-05/FAS-50, and the highest, FS-720/A40. This corresponds to an ohmic loss difference of approximately 0.125 V at 250 mA/cm^2^. While the total difference in polarization voltage is around 1 V, as shown in Figure 2, this suggests that ohmic losses account for only a portion of the observed voltage difference, and additional factors—such as kinetic or mass transport limitations. More specifically, overpotentials contributing to voltage losses may arise from local pH changes at the electrodes, which can reduce reaction rates, as well as from catalyst poisoning or other kinetic alterations. In Figure 3, where DI water is symmetrically fed, the HFRs are similar, which aligns with the comparable polarization performance observed under those conditions. It is evident that contaminant ions affect the HFR and polarization performance of the different BPMs in distinct ways.

The polarization curves and HFR data indicate that the membrane conductivity is reduced with the asymmetric feed, consistent with a decrease in electrolyzer performance. We attributed this reduction in conductivity in part to a change in mobility for the charge-carrying species with the introduction of the contaminant species. Na^+^ and Cl^–^ ions have lower mobilities in the membrane phase compared to H^+^ and OH^–^, resulting in a higher membrane resistance.^36,37^ The dilute-solution mobility for Na^+^ is 5.19×10^–8^ m^2^/(s V) versus 36.23×10^–8^ m^2^/(s V) for H^+^, while Cl^–^ is 7.91×10^–8^ m^2^/(s V) versus 20.64×10^–8^ m^2^/(s V) for OH^–^.^38^ Despite the differences in ion mobility among the ionic species, the membrane experiences additional conductivity losses compared to dilute aqueous solutions due to the physicochemical interactions between the polymer, water, and ions. In examining the (sulfonic acid-based) CELs, the conductivity of the membrane when contaminant alkali metal cations are introduced is influenced by the cation’s affinity to the sulfonic acid fixed charge groups, the water content in the membrane, and the water transference coefficients, as reported by Okada et al.^36^

Na^+^ has a greater affinity than H^+^ to sulfonic acid groups beyond the dilute solution mobilities in water.^32^ Multiple studies have shown that as the ratio of Na^+^ to fixed sulfonic acid groups increases, a reduction in water uptake occurs (osmotic deswelling), decreasing the ionic conductivity.^37,39,40^ The reduction of water content in perfluorinated membranes (all CELs tested were perfluorinated-based) alters their characteristic phase-separated morphology by decreasing water channel size and enhancing interactions between ions and the polymer matrix. This hinders ion transport and consequently reduces the conductivity.^37^

Similar arguments (ion affinity, water content, and water transference) of the conductivity losses in the CEL can also be applied to the AEL, with the Cl^–^ negatively affecting the conductivity, contributing to higher BPM resistances and cell voltages.

Marino et al.^41^ showed that for a poly­(arylene ether) AEM, the conductivity, water uptake, and degree of dissociation at a given relative humidity was lower in the Cl^–^ form versus the OH^–^ form. Yet our understanding of water and ion transport in AEMs is still nascent compared to CEMs.^38^

### Chlorine Species Crossover

Figure 4 shows the total Cl species crossover flux for each of the four BPM tested. The flux is reported as total Cl, which includes additional Cl species that could have been produced from the Cl^–^ oxidation reaction such as HOCl and OCl^–^. Marin et al.^20^ showed that after 6 h of BPM water electrolysis at 250 mA/cm^2^, there were minimal concentrations of free Cl species (e.g., around 0.22 μmol/L of Cl_2_, HOCl, and OCl^–^), meaning most of the Cl detected is likely in the Cl^–^ form. E98-05/FAS-50 had the least amount of total Cl crossover and the FS-720/A40 BPM demonstrated the highest amount of total Cl crossover among all four BPMs, with close to two orders of magnitude difference between the E98-05/FAS-50 and FS-720/A40. This demonstrates that the total Cl crossover is dictated by the properties of the CEL in the BPM.

**Figure 4:**
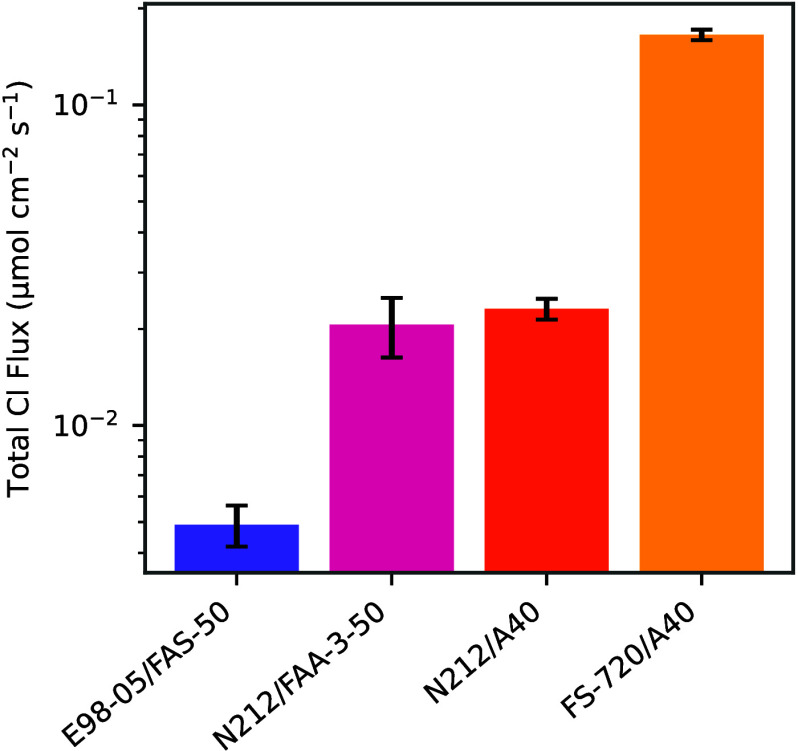
Total Cl crossover flux during BPM water electrolysis operation for four different BPMs, measured through ICP-MS. Flux was measured at a constant current density of 250 mA/cm^2^.

To assess the contribution that each membrane layer had on total Cl crossover, two of the BPMs fabricated had the same CEL (N212/FAA-3-50 and N212/A40) and two BPMs had the same AEL (N212/A40 and FS-720/A40). Changing the AEL layer had little effect on the crossover, as N212/FAA-3-50 and N212/A40 BPMs had similar crossover. However, the BPMs with dissimilar CELs (N212/A40 and FS-720/A40) had almost an order magnitude of difference in fluxes. This indicates that the CEL plays a more significant role in controlling total Cl crossover.

The effect of the CEL and AEL on total Cl crossover can be explained with Donnan equilibrium and ion exclusion principles. In this system, the CEL is adjacent to the 0.5 mol/L NaCl catholyte feed, preferentially allowing Na^+^ ions to enter the CEL. The Donnan equilibrium principle suggests that the concentration of Cl^–^ ions will be lower than that of cations in the CEL. Yet for Cl^–^ ions in the CEL, there are minimal impediments to the movement of Cl^–^ ions from crossing from the CEL’s membrane phase to the AEL’s, since the AEL facilitates the transport of anions. Consequently, it is the degree of anion exclusion from the CEL that determines the extent of total Cl crossover within this BPM system.

Hence, when choosing membranes for BPM applications with asymmetric feeds that include contaminant ions, it is crucial to evaluate the individual ion exchange layers for their ability to prevent the crossover of undesired ion species, e.g., salt permeability studies.^31,42^ In the case of an asymmetric feed, the layer facing the contaminated electrolyte feed will be important in controlling the performance of the electrolyzer.

### Anolyte pH Change

The H^+^ concentration in the anolyte reservoir was measured by the change in pH as a function of cell operation time as a function constant current density 250 mA/cm^2^. Using the pH data vs. time (Figure S1 and Figure S2) a H^+^ accumulation rate in the anolyte compartment was calculated. The positive H^+^ accumulation (Figure 5) rate indicates a decreasing pH in the anolyte.

**Figure 5:**
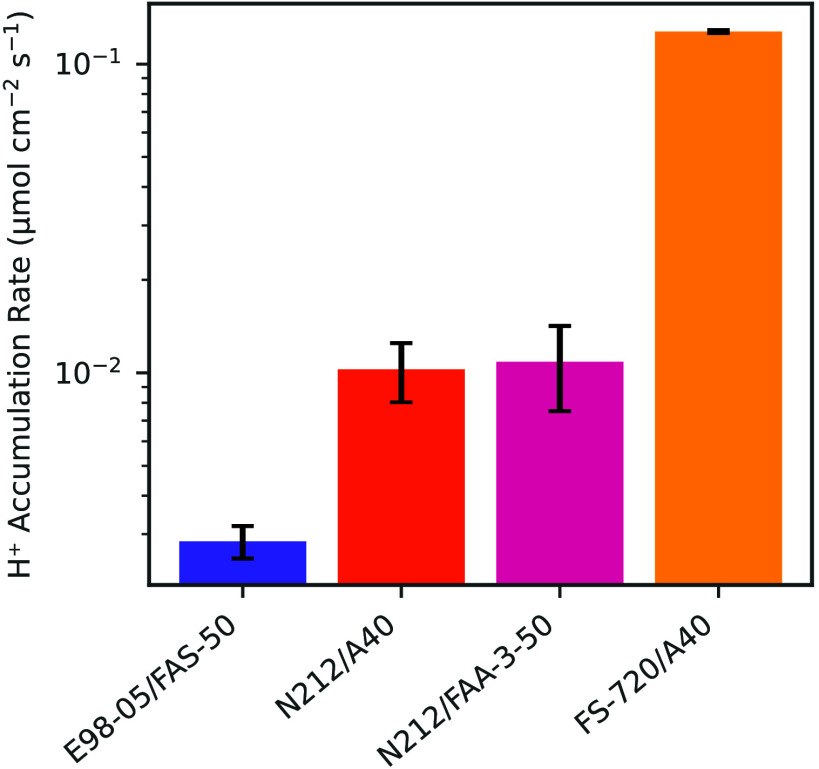
H^+^ accumulation rate in the anolyte during BPM water electrolysis operation for four different BPMs, measured through pH. pH was measured and the accumulation was computed at a constant current density of 250 mA/cm^2^.

While Cl^–^ ions are not excluded from entering the AEL and can migrate toward the anode under the applied electric field, their transport into the anolyte must be accompanied by a charge balancing mechanism to preserve electroneutrality in the bulk solution. In this system, the electrooxidation of water can serve this purpose, generating H^+^ and resulting in a net [H^+^] increase in the anolyte balanced by Cl^–^ flux across the membrane (Figure 6). Understanding this process is crucial, as acidification of the anolyte solution can reduce the efficiency of the electrolyzer.

**Figure 6:**
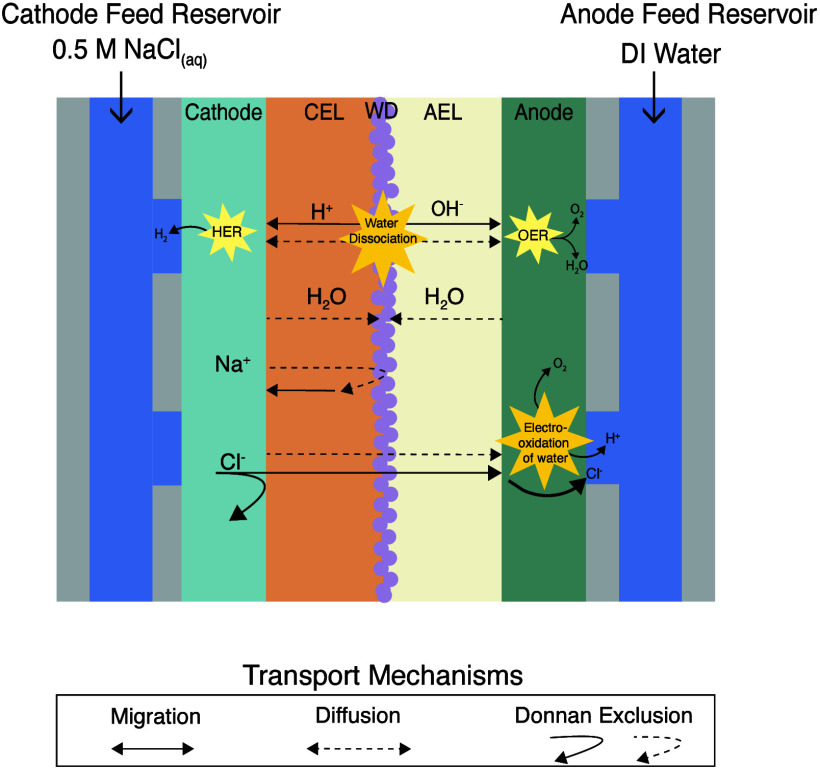
Schematic of ion and water transport in a BPM electrolyzer during water electrolysis, showing key transport mechanisms (migration, diffusion, Donnan exclusion) across the AEL and CEL. DI water and 0.5 M NaCl feed the anode and cathode, respectively. HER and OER occur at the cathode and anode.

The accumulation of one H^+^ ion in the anolyte for each Cl^–^ ion that crosses the membrane and enters the bulk anolyte is reflected in their similar values in the H^+^ accumulation in Figure 5 and the total Cl flux in Figure 4. E98-05/FAS-50 exhibited the lowest Cl crossover and lowest H^+^ accumulation rate, whereas FS-720/A40 demonstrated the highest in both metrics. N212/FAA-3-50 and N212/A40 recorded crossover and accumulation rates that were not only within error of each other but also intermediated between E98-05/FAS-50 and FS-720/A40.

## Conclusions

This study examined the water electrolysis performance for four different BPMs: E98-05/FAS-50, N212/FAA-3-50, N212/A40, and FS-720/A40. The work focused on how variations in saline electrolyte feed conditions (symmetric and asymmetric) influence the performance and ion crossover dynamics of each of the four BPMs.

Asymmetric 0.5 mol/L NaCl feed conditions (0.5 mol/L NaCl at the catholyte, DI water at the anolyte) led to higher operating cell voltages across all four BPMs compared to the operating cell voltages with symmetric DI water feeds. The differences in operating voltage within the ohmic overpotential region can be attributed to differences in mobility between the contaminant ion species, Na^+^ and Cl^–^, and H^+^ and OH^–^, and physicochemical interactions between the polymer, water, and ions.

Among the four different BPMs during electrolysis (at 250 mA/cm^2^) with symmetric feeds, the N212/A40 BPM had the lowest cell voltages (1.94 V) with increasing current density and FS-720/A40 had the highest voltages (2.17 V) However, with the asymmetric feed conditions, the E98-05/FAS-50 had the lowest cell voltages (3.11 V) while FS-720/A40 had the highest cell voltages (3.93 V).

Concerning Cl species crossover, E98-05/FAS-50 BPM had the lowest amount of crossover while FS-720/A40 had the highest. The crossover data indicates that the CEL has a greater effect on Cl crossover than the AEL. This is explained through Donnan exclusion and the degree to which the co-ions are excluded from the CEL since the 0.5 mol/L NaCl feed shares an interface with the CEL.

The polarization curves and the ion flux results indicate that when selecting membranes for BPM water electrolysis with asymmetric feeds, the ion transport properties of membranes are a crucial design parameter. The ion transport properties of the membrane influence both electrolyzer performance and the rate of unwanted reactions, such as Cl oxidation. Future work should investigate the multi-ionic transport properties of BPMs, especially with asymmetric feeds, which would offer insight into the performance of systems operating with seawater or other natural feed sources. Additional future studies could explore the permselectivity of the individual layers within the BPM to better understand their contributions to overall membrane performance. Such investigations, while beyond the scope of the present work, would provide valuable insights into optimizing BPM design.

## Supplementary Material



## Data Availability

All raw data employed for analysis and visualization in this work are publicly available on Zenodo at https://zenodo.org/records/14904520.

## Notes

Plot of pH versus time during a water electrolysis trial with asymmetric feed and plot of calculated H^+^ concentration versus time during a water electrolysis trial (PDF)

## ⊥

Department of Chemical Engineering and Materials Science, Michigan State University, East Lansing, Michigan 48824-1312, United States

## #

Energy Technologies Area, Lawrence Berkeley National Laboratory, Berkeley, California 94720-8099, United States.
