# Supplementary material for: Controlling Chloride Crossover in Bipolar Membrane Water Electrolysis
Source: ACS Electrochem. 2025 Jul 16;1(9):1812–20. doi: 10.1021/acselectrochem.5c00175 (PMC12415932; doi:10.1021/acselectrochem.5c00175)
Supplement: Supplementary file 1 [file ec5c00175_si_001.pdf]

Supporting Information: Controlling Chloride Crossover in Bipolar Membrane Water Electrolysis

Maria F. Rochow,[†,‡] Daniela H. Marin,[¶,§] Harrison J. Cassady,[‖,⊥] Ryan T. Hannagan,[¶,§] Katherine Yan,[¶,§] Joseph T. Perryman,[¶,§] Adam C. Nielander,[¶,§] Thomas F. Jaramillo,[¶,§] and Michael A. Hickner[*,†,‡]

†*Department of Material Science and Engineering, Penn State, University Park, PA 16802*

‡*Current Affiliation: Department of Chemical Engineering and Materials Science, Michigan State, East Lansing, MI 48824-1312*

¶*Department of Chemical Engineering, Stanford University, Stanford, CA 94305*

§*SUNCAT Center for Interface Science and Catalysis, SLAC National Accelerator Laboratory, Menlo Park, CA 94025*

‖*Department of Chemical Engineering, Penn State, University Park, PA 16802*

⊥*Current Affiliation: Energy Technologies Area, Lawrence Berkeley National Laboratory, Berkeley, CA 94720-8099*

E-mail: mhickner@msu.edu

Contents

List of Figures **S-2**

List of Figures

S1 pH change in the anolyte container during BPM water electrolysis. S-3

S2 H^+ concentration produced during water electrolysis. S-4

This supplemental document provides additional figures to support and expand upon the findings presented in the main manuscript. Figure S1 presents a graph of pH versus time for one of the BPM trials conducted during water electrolysis with an asymmetric feed. Figure S2 is the calculated H^+ concentration from the data in Figure S1.

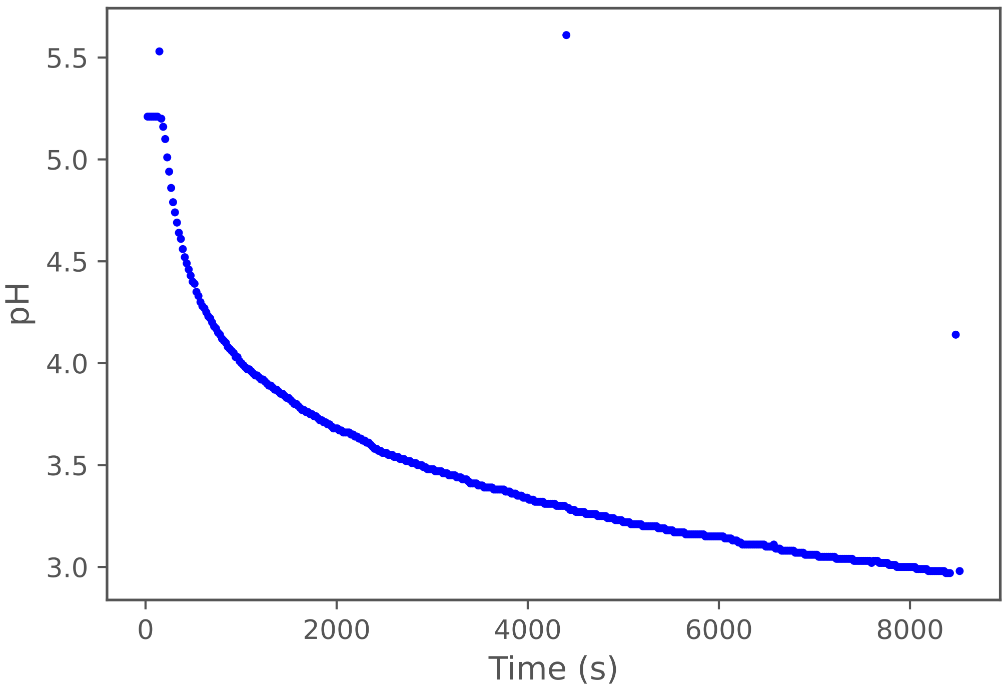

Figure S1: pH change in the anolyte container during a BPM water electrolysis trial using the Fumasep FS-720/PiperION (FS-720/A40) membrane. pH was measured at a constant current density of 250 mA/cm^2.

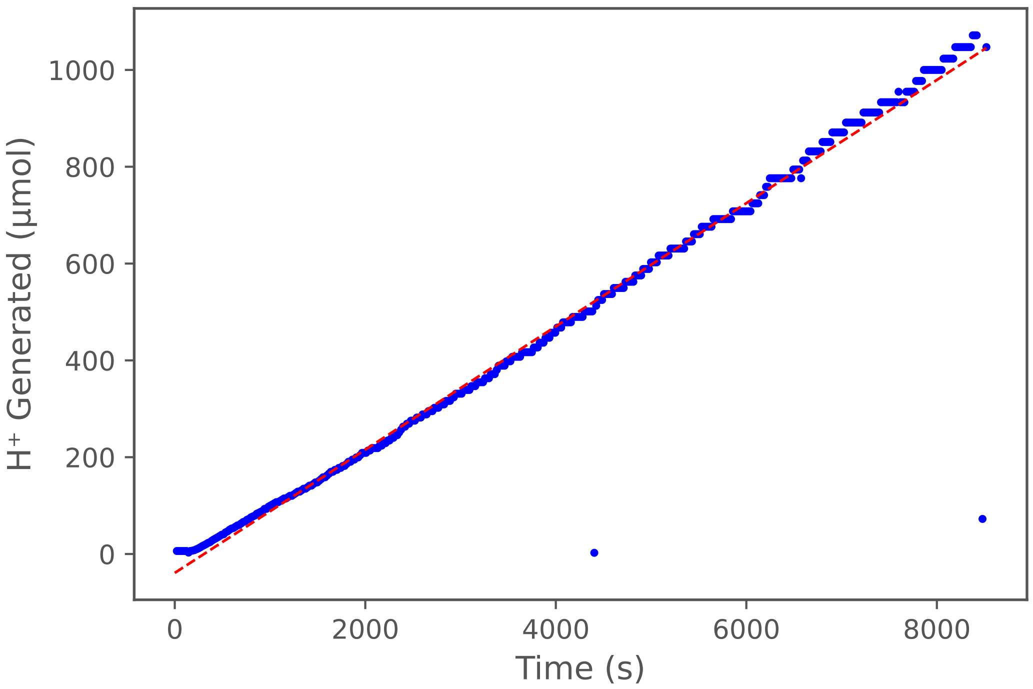

Figure S2: The H^+ concentration produced, derived from pH measurements (Figure S1), in the anolyte container during water electrolysis with the Fumasep FS-720/PiperION (FS-720/A40) membrane. The red dashed line represents the linear fit from the linear regression performed to calculate the H^+ accumulation rate.
